# Anti-Inflammatory Diet and Probiotic Supplementation as Strategies to Modulate Immune Dysregulation in Autism Spectrum Disorder

**DOI:** 10.3390/nu17162664

**Published:** 2025-08-18

**Authors:** Carlos Andrés Naranjo-Galvis, Diana María Trejos-Gallego, Cristina Correa-Salazar, Jessica Triviño-Valencia, Marysol Valencia-Buitrago, Andrés Felipe Ruiz-Pulecio, Luisa Fernanda Méndez-Ramírez, Jovanny Zabaleta, Miguel Andres Meñaca-Puentes, Carlos Alberto Ruiz-Villa, Marcela Orjuela-Rodriguez, Juan Carlos Carmona-Hernández, Luisa Matilde Salamanca-Duque

**Affiliations:** 1 Facultad de Salud, Universidad Autónoma de Manizales, Antigua estación del Ferrocarril, Manizales 170004, Colombia; 2 Facultad de Ciencias de la Salud, Universidad de Manizales, Manizales 170004, Colombia; 3 Facultad de Ciencias de la Salud, Universidad Católica de Manizales, Manizales 170004, Colombia; 4 Department of Interdisciplinary Oncology, Louisiana State University Health Sciences Center, New Orleans, LA 70112, USA; 5 Facultad de Inteligencia Artificial e Ingeniería, Universidad de Caldas, Manizales 170004, Colombia; 6 Centro de Bioinformática y Biologia Computacional de Colombia (BIOS), Manizales 170004, Colombia; 7 Grupo de Investigación Médica, Universidad de Manizales, Manizales 170004, Colombia

**Keywords:** autism spectrum disorder, anti-inflammatory diet, probiotics, cytokines, immune modulation, nutritional intervention

## Abstract

**Background/Objectives**: Autism spectrum disorder (ASD) is a neurodevelopmental condition associated with behavioral and cognitive impairments. Increasing evidence also links ASD with systemic immune dysregulation, including abnormal cytokine profiles and chronic low-grade inflammation. Emerging evidence suggests that targeted dietary strategies and probiotic supplementation may modulate immune responses and gut–brain interactions in patients with ASD. This study aimed to evaluate the immunomodulatory effects of a structured anti-inflammatory diet (*NeuroGutPlus*) compared to multi-strain probiotics in children with ASD. *NeuroGutPlus* is a nutritionally complete anti-inflammatory dietary protocol that targets gut integrity, inflammation, and mitochondrial function. It includes a diet low in gluten, FODMAPs, casein, and artificial additives, and a high intake of omega-3 fatty acids, polyphenols, and fermentable fibers. **Methods**: A total of 30 children with ASD and 12 neurotypical controls were enrolled in a 12-week randomized controlled nutritional trial. Participants received either a *NeuroGutPlus* anti-inflammatory diet, probiotic supplementation (16 strains of *Lactobacillus* and Bifidobacterium), or no intervention. Plasma levels of 20 cytokines and chemokines were measured pre- and post-intervention using multiplex Luminex immunoassays. Principal component analysis (PCA) was used to explore shifts in the immune profile. **Results**: Patients treated with the *NeuroGutPlus* diet significantly reduced IFN-γ levels (*p* = 0.0090) and showed a stabilizing effect on immune profiles, as evidenced by PCA clustering. Probiotic supplementation led to a significant increase in IL-8 (+66.6 pg/mL; *p* = 0.0350) and MIP-1β (+74.5 pg/mL; *p* = 0.0100), along with a decrease in IFN-γ (*p* = 0.0070), suggesting reconfiguration of innate immune responses. Eight out of eleven biomarkers showed significant post-intervention differences between groups, indicating distinct immunological effects. **Conclusions**: This study provides evidence that anti-inflammatory diets exert broader and more consistent immunoregulatory effects than probiotics alone in children with ASD. These findings support the inclusion of precision dietary strategies as non-pharmacological interventions to mitigate immune-related dysfunction in patients with ASD.

## 1. Introduction

Autism spectrum disorder (ASD) is a neurodevelopmental condition characterized by persistent impairments in social communication and interaction, along with restricted and repetitive patterns of behavior, activities, or interests, as defined in the DSM-5 (Diagnostic and Statistical Manual of Mental Disorders, Fifth Edition) [1]. Recent global estimates indicate that ASD affects approximately 0.76% of children, with a higher reported prevalence in high-income countries, which may be influenced by diagnostic resources and access to specialized health services [2,3].

Beyond the intrauterine development stage, individuals with ASD often exhibit a dysregulated immune response characterized by excessive inflammation and heightened oxidative stress, which appears to persist throughout their lives. This condition is linked to the severity of core symptoms, including challenges in reciprocal communication, social interactions, and repetitive behaviors. Research, such as the study by Yin et al., has demonstrated an atypical inflammatory response, with levels of IL-6, IL-1β, IL-12p70, MIF, eotaxin-1, MCP-1, IL-8, IL-7, IL-2, IL-12, TNF-α, IL-17, and IL-4 differing significantly from those in neurotypical individuals [4]. Ashwood et al. [5] on the other hand, reported significant increases in IL-1β, IL-6, IL-8 and IL-12p40 levels, while Zhao et al. [4] showed increased levels of IL-6, IL1β. More recently, Aldossari et al. [6] showed immunological abnormalities with imbalances in cytokine production, in the expression of chemokine receptors, overexpression of CD40, and high levels of pro-inflammatory cytokines such as IFN-γ, TNF-α, and IL-17A. All the previous information has served to propose different interventions that aim to influence ASD pathology in light of the relationship between chronic inflammation, dysregulated immune responses, and the gut–microbiota–brain axis.

The “gut–brain axis,” a two-way communication network connecting the digestive system with the central nervous system, has become a significant factor in the pathophysiology of ASD. In ASD, increased levels of pro-inflammatory cytokines can affect the brain either directly by crossing the blood–brain barrier, or indirectly, resulting in neuroinflammation and changes in neural connectivity. Concurrently, many children with ASD present with gastrointestinal symptoms, which are believed to exacerbate behavioral symptoms through microbial dysbiosis and immune activation [7,8,9].

Children with ASD often exhibit poor feeding practices due to selective eating behaviors. Combined with socioeconomic factors, this results in diets low in vegetables, fruits, and protein intake [10]. Evidence shows a high consumption of foods rich in pro-inflammatory compounds, which increase the levels of circulating cytokines at the peripheral and central levels, impact neurodevelopment, and correlate with the intensification of symptoms [11]. In addition, since diet is one of the main environmental determinants affecting the microbiota composition, these intake restrictions may play a significant role in gut microbiota diversity. The latter is supported by evidence that denotes marked differences between populations with different geographical locations and diets, as well as the effect of short-term changes in the microbiota from studies with dietary interventions [12,13].

Although inflammatory pathways are implicated in ASD, novel therapeutic strategies are being developed to attenuate their effects, including the use of natural anti-inflammatory compounds [14]. The main nutrients that may affect the nutritional management of ASD include omega-3 fatty acids and probiotics, as they can modulate the gut microbiota and influence symptoms; however, further clinical research is required to confirm this [15]. Research has indicated the necessity of addressing nutritional deficiencies in individuals with ASD through the supplementation of omega-3 fatty acids, probiotics, vitamins, and minerals, alongside medical and psychological interventions. Appropriately designed and individualized diets may also mitigate the symptoms of autism and reduce the incidence of gastrointestinal disorders. Consequently, it is imperative for parents and caregivers to recognize the advantages of nutritional therapy and the importance of monitoring treatment in patients with ASD [16,17].

Siafis et al. [18] published a meta-analysis on pharmacological interventions and dietary supplements for the treatment of ASD, finding indications of improvement with carnosine, haloperidol, folic acid, guanfacine, fatty acids such as omega-3, probiotics, sulforaphane, tideglusib and valproate; however, the evidence was limited and not statistically significant. In contrast, Li et al. [19] reported that abnormal *Clostridium* counts associated with gastrointestinal disorders improve when treated with antibiotics for other pathologies or when potential next-generation probiotics such as *Bacteroides fragilis* are administered.

Scientific evidence suggests that chronic inflammation may play a significant role in autism symptomatology, including behavioral, communication, and social skills [20]. Thus, it is evident that non-pharmacological interventions, such as dietary strategies and probiotic supplements used in ASD interventions, become relevant in the face of growing evidence that nutritional and immunological factors can significantly influence the symptomatology of ASD [21]. Supplementation with mixtures of beneficial bacteria such as Lactobacillus, Bifidobacterium, Akkermansia, and *Prevotella* has shown multiple benefits, including reduction in gastrointestinal symptoms such as constipation, diarrhea, and abdominal pain; improvement in behavioral symptoms related to emotional regulation and attention; positive modification of intestinal microbial composition, with increased bacterial diversity; stimulation of the production of short-chain fatty acids (SCFAs) with neuroprotective effects; and suppression of the expression of pro-inflammatory cytokines, contributing to the regulation of the immune system [22,23].

At the same time, various dietary interventions have been explored in children with ASD. The most studied are gluten- and casein-free diets (GFCF)**;** ketogenic diets, used for their impact on brain function; diets rich in fiber, focused on promoting the growth of beneficial bacteria; and a Mediterranean diet, high in polyphenols and antioxidants, which has anti-inflammatory and neuroprotective effects [24,25]. However, potential risks have been identified, including nutritional deficiencies (e.g., B-complex vitamins, iron, fiber), reduction in beneficial gut bacteria, proliferation of opportunistic pathogens, gastrointestinal side effects (constipation, diarrhea, vomiting), and altered lipid metabolism [26].

Although there is no standardized definition of an anti-inflammatory diet in international nutrition guidelines, the term commonly refers to a diet containing foods that help prevent inflammation and limiting those that cause it. This means that the approach to our anti-inflammatory diet meets the criteria of foods that improve intestinal transit by providing insoluble fiber in adequate amounts as required, as well as micronutrients including vitamins and minerals for which scientific evidence supports an anti-inflammatory effect, and which were evaluated in terms of their consumption and contribution to the current research. These micronutrients are iron, selenium, magnesium, zinc, omega-3 fatty acids, vitamin B1 thiamine, B2 riboflavin, B3 niacin, B9 folic acid, vitamin A, vitamin C, and vitamin E. For macronutrients, their contributions were guided according to the Recommended Intakes of Essential Nutrients (RIEN) (*Energy and Nutrient Intake Recommendations*) established for the Colombian population, in addition to the consumption of fiber and water. In contrast, a decrease in the consumption of foods that could have an inflammatory effect in situations of increased intestinal permeability, such as gluten and FODMAPs (*Fermentable Oligosaccharides*, *Disaccharides*, *Monosaccharides*, *and Polyols*), was implemented.

The findings of this study indicate that specific nutritional interventions, such as dietary adjustments or the inclusion of supplements, could play a significant role in the management of core and associated symptoms in individuals with ASD. This perspective opens new avenues for therapeutic strategies that complement traditional interventions, emphasizing the importance of a comprehensive approach, in which nutrition is emerging as a critical component that deserves greater attention and exploration in the context of global interventions aimed at improving the health, well-being, and quality of life of this population. In the present study, three structured instruments were used as part of the initial evaluation process and clinical–nutritional follow-up of the study on dietary interventions in children with ASD.

The *NeuroGutPlus* diet is an anti-inflammatory and FODMAPs-controlled intervention designed for children with ASD. It includes foods such as fruits and vegetables (rich in antioxidants), olive oil, walnuts, almonds, peanuts, seeds, and fish (sources of omega-3s), which help reduce chronic inflammation. On the other hand, it limits pro-inflammatory foods such as sugar, saturated fats, and fried and processed foods (deli meats, commercial juices, sodas, packaged foods), which can alter the intestinal microbiota related to the immune and nervous systems (it also restricts FODMAPs to relieve gastrointestinal overload).

Interventions aimed at restoring microbial balance, such as probiotics and specialized diets, have gained popularity. However, evidence of their comparative efficacy in modulating immune markers remains scarce. This study addresses this gap by comparing the effects of a comprehensive dietary intervention (*NeuroGutPlus*) with standard probiotics on immune biomarkers in children with ASD.

The primary objective was to evaluate immunomodulatory effects, defined as measurable changes in plasma cytokine and chemokine concentrations following 12 weeks of intervention. The primary endpoint was the Δ (POST-PRE) concentration of cytokines and chemokines, while the secondary endpoint involved exploratory multivariate analysis (PCA) to assess global immune network reorganization. Behavioral outcomes were not included in this analysis and are acknowledged as a limitation.

## 2. Materials and Methods

### 2.1. Study Design and Participants

**Figure 1:**
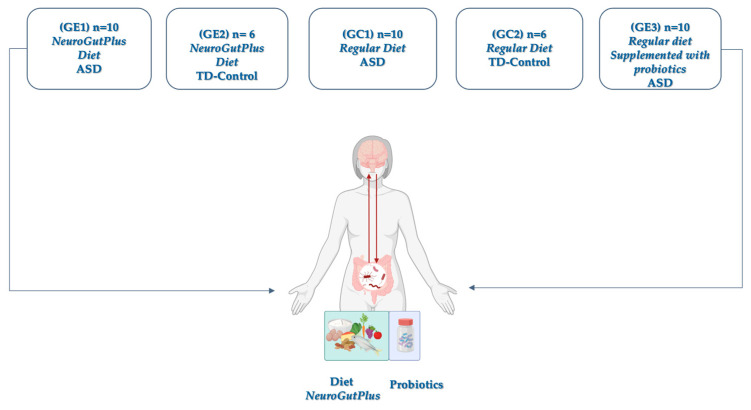
**Study design illustrating dietary and probiotic interventions across ASD and control groups**. **GE1**—children diagnosed with ASD who received a diet designed under the principles of inflammatory restriction (NeuroGutPlus). **GE2**—neurotypical controls who also received an anti-inflammatory diet NeuroGutPlus. **GC1**—control group with ASD consuming their usual diet without modifications. **GC2**—neurotypical control group fed a conventional diet. **GE3**—participants with ASD who received their usual diet along with probiotics such as Lactobacillus and Bifidobacterium.

A total of 30 children (ages 6–17) diagnosed with ASD based on the DSM-5 criteria and 12 typically developing (TD) controls were enrolled in a 12-week controlled trial. Only children with high-functioning autism (Level I, DSM-5) and active enrollment in the Colombian General System of Social Health Insurance (SGSSS) were recruited from therapeutic support institutions to guarantee the representativeness of the Colombian coffee-growing region. Criteria were confirmed ASD diagnosis with stable behavioral therapy for at least 3 months, and no antibiotic or immunosuppressive use within the last month. The design included five groups, including experimental and control groups (Figure 1).

### 2.2. Intervention Protocols

Each group was evaluated within the framework of the TCAC-2018 guidelines (Colombian Table of Feeding by Cycles) and the Energy and Nutritional Intake Requirements (*RIEN*), allowing homogeneous control of the food intervention in terms of nutritional quality and caloric adequacy. This design made it possible to compare not only the differential effect of the anti-inflammatory diet versus the regular diet but also the specific effect of probiotic supplementation on various neurological profiles. The inclusion of neurotypical children in the control group reinforced the comparative nature of the study and provided a baseline for immunological analysis.

*NeuroGutPlus* is a nutritionally complete anti-inflammatory dietary protocol that targets gut integrity, inflammation, and mitochondrial function. It includes a diet low in gluten, FODMAPs, casein, and artificial additives, and a high intake of omega-3 fatty acids, polyphenols, and fermentable fibers. This was delivered in food packages to each family, who were trained in their preparation and distribution. The *Probiotic* group received a daily dose of a multi-strain probiotic supplement (*Lactobacillus* and *Bifidobacterium* spp.) for the same duration.

One branch of the intervention included children with and without ASD under a regular diet supplemented with probiotics (registered under INVIMA code SD2020-0004514) within the clinical–nutritional study. The probiotic supplement was administered in a bottle containing 30 capsules of hard gelatin, following the dosage indicated by the clinical protocol. This multi-strain probiotic was selected for its safety, efficacy, and microbiological stability. The formula includes a combination of 16 probiotic strains, grouped into two genera widely studied for their beneficial effects on gut and immune health: **Genus** Lactobacillus: *L. acidophilus, L. plantarum, L. casei, L. paracasei, L. bulgaricus, L. brevis, L. reuteri, L. salivarius, L. fermentum, L. gasseri, and L. rhamnosus.* **Genus** *Bifidobacterium*: *B. lactis, B. infantis, B. bifidum, B. breve, and B. longum*. These were selected based on evidence supporting their immunomodulatory potential, gut barrier restoration, and relevance to *the microbiota–gut–brain axis in ASD. **Lactobacillus rhamnosus** and **Bifidobacterium longum**, among other strains in the blend, have been associated with decreased gut permeability, reduced systemic inflammation, and improved gastrointestinal function. Multi-strain combinations are recommended by recent meta-analyses as they provide complementary metabolic and immune functions, enhancing overall therapeutic impact* [8,27,28,29].

These strains have demonstrated anti-inflammatory properties (↓ TNF-α, IL-6), immunomodulatory effects (↑ IL-10, TGF-β), restoration of the intestinal barrier (improvement of epithelial integrity and tight junctions), reduction in dysbiosis, and normalization of the microbiota–gut–brain axis.

Adherence to the *NeuroGutPlus* dietary intervention was monitored using a multi-step strategy: (i) caregivers completed weekly structured food logs documenting compliance with recommended food groups and avoidance of restricted foods; (ii) weekly telephone calls with a registered dietitian were conducted to address challenges and reinforce compliance; and (iii) unused food items were verified during follow-up visits. Based on these measures, adherence was estimated at >85% for the dietary group.

### 2.3. Sample Collection and Immune Profiling

Peripheral blood samples were obtained at two time points: baseline (PRE) prior to intervention initiation, and POST-intervention after 12 weeks. To control for circadian and nutritional variability, all samples were collected in the morning (7:30–9:30 a.m.) following an overnight fast. Standardized venipuncture procedures were used across all participants. Samples were processed within 2 h and stored at −80 °C until cytokine analysis was conducted via a multiplex Luminex assay. The ProcartaPlex^TM^ Human Th1/Th2 Cytokine & Chemokine Panel 1 20plex enables the exploration of immune function by analyzing 20 protein targets in a single well using the MAGPIX detection system (Luminex Corp., Austin, TX, USA) (Cat. No. EPX450-12171-901). The panel allows the detection of *Th1/Th2* cytokines (GM-CSF, IFN-γ, IL-1β, IL-2, IL-4, IL-5, IL-6, IL-8, IL-12p70, IL-13, IL-18, TNF-α), chemokines, including Eotaxin (CCL11), GRO-α (CXCL1), IP-10 (CXCL10), MCP-1 (CCL2), MIP-1-α (CCL3), MIP-1β (CCL4), RANTES (CCL5), and SDF-1α, following the manufacturer’s instructions (Thermo Fisher Scientific, Waltham, MA, USA). Duplicate plasma samples were analyzed before and after the intervention with the *NeuroGutPlus* anti-inflammatory diet and compared to probiotics. The results presented included markers with pre- and post-intervention detection thresholds.

## 3. Statistical Analysis

All statistical analyses were conducted in Python 3.x using the SciPy library (SciPy. stats module) for statistical tests including Shapiro–Wilk normality testing, Levene’s test for homogeneity of variance, paired and independent *t*-tests, Wilcoxon signed-rank test, and Mann–Whitney U test. Data manipulation and preprocessing were performed using pandas with numerical computations supported by NumPy. The comparative analysis between pre- and post-intervention measurements employed a hierarchical decision framework to select the most appropriate statistical test according to the data characteristics and underlying assumptions.

Statistical test selection was based on systematic evaluation of normality (Shapiro–Wilk test, α = 0.05) and homogeneity of variances (Levene’s test, α = 0.05) for each biomarker. Parametric tests were prioritized when assumptions were met due to their superior statistical power compared to non-parametric alternatives. Changes in statistical tests between PRE and POST analyses reflect differences in data distribution following interventions. For cases where pre- and post-groups exhibited equal sample sizes, individual participant identifiers were verified to confirm proper pairing of observations across time points.

The initial assessment determined whether the data could be analyzed in a paired-sample design by evaluating sample size equality and confirming a minimum of two observations per group. When paired analysis was feasible, the normality of difference scores (PRE–POST) was assessed using the Shapiro–Wilk test. Normally distributed differences were analyzed using paired *t*-tests, whereas non-normal differences were evaluated using the Wilcoxon signed-rank test, a non-parametric alternative that does not assume normality. For unpaired data configurations, the analysis proceeded through sequential evaluation of distributional assumptions.

The normality of both pre- and post-groups was independently assessed using the Shapiro–Wilk test. When both groups satisfied normality assumptions, the homogeneity of variances was evaluated using Levene’s test. Equal variances supported the application of Student’s independent *t*-test, whereas unequal variances necessitated Welch’s *t*-test, which adjusts for heteroscedasticity. When normality assumptions were violated in either group, the Mann–Whitney U test was employed as a non-parametric alternative. PCA was performed using Scikit-learn, with data processing handled via Pandas and NumPy, and visualizations generated using Seaborn (v0.13.2) and Matplotlib (v3.10.3).

This methodological approach ensures statistical validity by matching analytical techniques to data characteristics, thereby maintaining appropriate Type I error rates while maximizing statistical power. Parametric tests were preferentially selected when distributional assumptions were satisfied, as they provide greater statistical power and more precise parameter estimates than their non-parametric counterparts. The systematic evaluation of assumptions prevents the misapplication of parametric tests when their underlying conditions are not met, thereby ensuring robust and reliable statistical inferences.

## 4. Results

### 4.1. Sociodemographic Characteristics of Study Participants

According to the sociodemographic analysis, the average age was 10.8 years in the ASD group and 11.8 years in the neurotypical group, with no statistically significant differences between groups (ANOVA, *p* = 0.45). The distribution by sex was 77% boys and 23% girls in total (as usual in ASD, an approximately 4:1 ratio). The groups did not differ significantly in sex proportion (chi-square = 0.38, *p* = 0.54) when comparing ASD vs. TD; both had a male majority. Regarding healthcare access, 17% of the participants did not receive interventions, and 83% of them received current health interventions such as general medicine, specialized medicine, physiotherapy, speech therapy, occupational therapy, and psychology.

A relevant finding was the low level of physical activity among participants with ASD: more than 60% did not exercise, compared to the TD group, where more than 60% exercised between 2 and 6 times a week. Lack of physical activity in ASD can have repercussions on physical, emotional, and metabolic health, which are key aspects for intervention. In total, 83% of the participants with ASD reported that they were receiving medical and psychological care, which shows a high degree of linkage with specialized services. The remaining 17% were not in an active intervention, which represents a priority group for the monitoring and strengthening of strategies for access to health. This analysis contributes to a comprehensive understanding of the individual and lifestyle factors that may have influenced the effectiveness of the nutritional and immunological interventions evaluated in this study.

Prior to the implementation of the nutritional intervention in this study, none of the participants had received any form of structured dietary or nutritional support. A comparison of nutritional status according to anthropometric indicators of height-for-age between children with ASD and neurotypical children (TD) was made. This parameter allows the detection of linear growth delays and is considered an indirect marker of long-term nutritional health status. In the control group, 92% of the children had an appropriate height for their age, 8% were at risk of delay, and 0% had values below the expected standard for their age. This suggests a relatively favorable nutritional profile in this population, possibly associated with better access to health services and a balanced diet.

In contrast, the ASD group showed a less favorable pattern with 78% of the children having adequate height, 17% being at risk of stunting, and 5% having a short height-for-age, which indicates stunting. These results reveal a greater nutritional vulnerability in the ASD population, which may be related to multiple factors, such as food restrictions (sensory or behavioral), gastrointestinal disorders (constipation, malabsorption), chronic low-grade inflammation, drug interactions that affect appetite or metabolism, and difficulties in accessing a balanced diet.

### 4.2. Immune Profile Changes

A comprehensive multiplex panel was used to measure plasma concentrations of Th1 and Th2-associated cytokines and chemokines. The present study evaluated the effect of dietary and probiotic interventions on inflammatory markers in children with ASD compared with neurotypical controls. The experimental design included two groups of neurotypical children (control and diet), and three groups of children with ASD (control, diet, and probiotics).

Cytokine profiling was performed using the ProcartaPlex™ Human Th1/Th2 Cytokine & Chemokine Panel 1 20plex (Cat. No. EPX450-12171-901) based on the Luminex xMAP^®^ technology. However, certain cytokines exhibited concentrations below the detection limit and were excluded from subsequent statistical analyses. This low detectability may be attributed to the limited expression of these molecules in the plasma compartment under physiological conditions, or to the specific inflammatory profile of the study population. The measurements were carried out at baseline (prior to the intervention, PRE), and POST-intervention, analyzing 11 cytokines and chemokines related to inflammatory processes expressed in pg/mL.

Baseline assessments revealed consistent cytokine levels across groups, with only monocyte chemoattractant protein (MCP1) exhibiting significant differences between the study groups (*p* = 0.004). In particular, the highest levels of this cytokine were observed in neurotypical children who received the dietary intervention. This finding suggests that at baseline, inflammatory markers were in similar ranges among all participants, providing a solid basis for evaluating the effects of subsequent interventions. Other biomarkers such as IL-8, MIP-1β, Eotaxin and SDF-1α showed variations in average levels between subgroups, but did not reach statistical significance, which could reflect biological heterogeneity (Table 1).

Table 2 shows the mean levels of immunological biomarkers in the subgroups of children with ASD after nutritional interventions, comparing the intragroup effects. Statistical tests (Kruskal–Wallis or ANOVA) were applied to detect significant differences between subgroups in the post-treatment phase.

### 4.3. POST-Intervention Cytokine and Chemokine Shifts

After nutritional intervention, the inflammatory landscape had remarkable changes. Eight of the eleven markers evaluated showed significant differences between the groups, contrasting markedly with initial homogeneity. Interleukin-8 (IL-8) showed a considerable increase in magnitude, rising from 2.9 pg/mL at baseline to 69.5 pg/mL POST-intervention, with a statistical significance of *p* = 0.035, indicating a potential activation of the innate immune response. Although IL-8 is associated with inflammation, some studies have reported an increase in the compensatory or immunomodulatory response induced by certain probiotics. MIP-1α increased significantly in the probiotic group (17.309 pg/mL), compared to the diet (4.367 pg/mL) and ASD Control (3.529 pg/mL) (*p* = 0.003), which may reflect chemokine activation after microbial intervention. IP-10 in the probiotic group showed higher levels (8.361 pg/mL) (*p* = 0.007) than in the other subgroups, suggesting a Th1 immune axis response, possibly related to microbiome modulation.

Eotaxin levels were also higher in the probiotic group, which could be related to immunoregulatory changes that affect eosinophils and antigen-presenting cells (*p* = 0.010). RANTES levels increased post-intervention in the probiotic group (11.401 pg/mL; *p* = 0.006) compared to other groups, suggesting a role in the recruitment of regulatory T cells or NK cells, and suggesting an immune rebalancing process. Although the absolute levels of IFN-γ remained low, there was a pattern of increase in the intervention groups, suggesting a moderate activation of adaptive immunity (*p* = 0.03). MIP-1β was significantly elevated in the probiotic group (199.603 pg/mL) (*p* = 0.001), which may be associated with the reactivation of cellular immunity and monocyte/macrophage chemoattraction. Finally, GRO-α showed an increase in the probiotic group (11.925 pg/mL) (*p* = 0.017), possibly related to the inflammatory processes regulated by the microbiota. SDF-1α (*p* = 0.504), MCP-1 (*p* = 0.06), and IL-18 (*p* = 0.344) levels were not significantly different between the post-intervention subgroups. This greater heterogeneity in the post-intervention inflammatory response indicates that different nutritional strategies generated distinctive immune response patterns, suggesting specific mechanisms of action for each type of intervention.

Mean difference tests were conducted to evaluate the effects of the *NeuroGutPlus* anti-inflammatory diet and probiotics on cytokine and chemokine levels in children with ASD and neurotypical children, based on PRE- and POST-intervention measurements.

**Figure 2:**
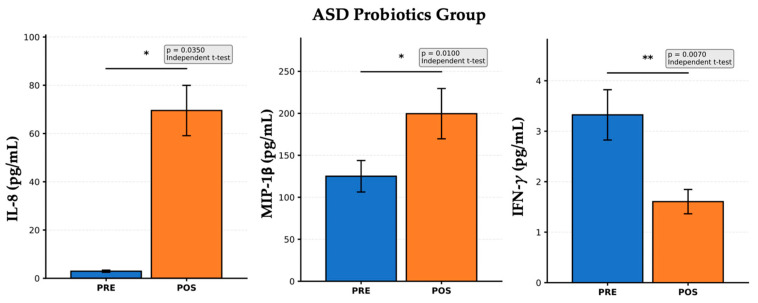
Changes in cytokine levels before and after probiotic intervention in children with ASD. Statistical markers were interpreted as * *p* < 0.05, ** *p* < 0.01. Each bar graph represents the mean concentration (±standard error) of a specific biomarker before (PRE) and after (POST) intervention.

Figure 2 shows a within-group comparison of the three immune biomarkers IL-8, MIP-1β, and IFN-γ, measured before (PRE) and after (POST) probiotic supplementation in children with ASD. A significant increase in IL-8 levels was observed after the probiotic intervention (*p* = 0.0350). IL-8 is a pro-inflammatory cytokine involved in neutrophil recruitment and activation. This increase may reflect an adaptive immune response mediated by the reshaped gut microbiota, potentially indicating improved immunosurveillance, rather than pathological inflammation. A statistically significant increase in MIP-1β levels was also observed (*p* = 0.0100). MIP-1β plays a role in attracting monocytes, macrophages, and lymphocytes to the sites of immune activation. This increase suggests immune adjustment or mobilization, possibly representing a rebalancing of the immune cell communication following microbial modulation. A significant reduction in IFN-γ levels was detected post-intervention (*p* = 0.0070). IFN-γ is a key Th1 cytokine associated with chronic inflammation and immune activation. This decrease indicated the downregulation of pro-inflammatory signaling, supporting the immunoregulatory effect of probiotic intervention in ASD. These findings are consistent with prior studies reporting that probiotics can modulate immune function in children with ASD, shifting the cytokine milieu towards a more regulated and balanced state. This may have implications for reducing neuroinflammation and improving behavioral outcomes.

**Figure 3:**
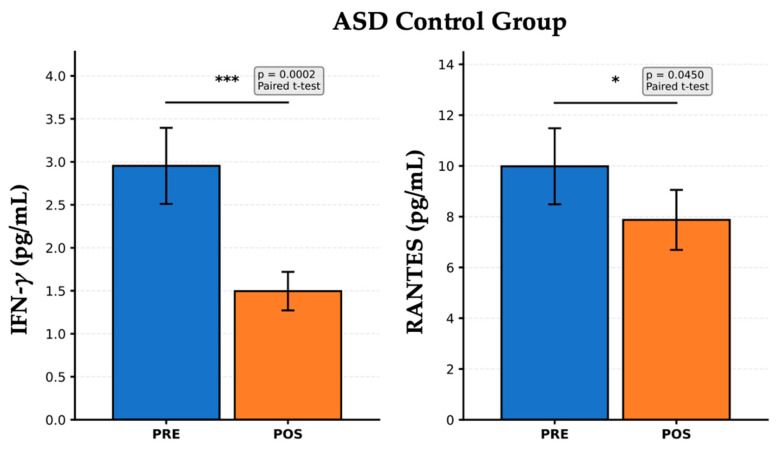
Reduction in IFN-γ and RANTES levels after standard care in the ASD Control group. Statistical markers were interpreted as * *p* < 0.05, *** *p* < 0.001.

Figure 3 presents the PRE- and POST-intervention cytokine levels in the ASD Control group, which did not receive probiotic supplementation, but may have undergone standard care or no dietary modulation. A significant reduction in IFN-γ levels was observed after the follow-up period (*p* = 0.0002). IFN-γ is a key cytokine associated with Th1 immune response and systemic inflammation. This marked decrease may reflect spontaneous variability, possible environmental changes, or a natural course of disease. However, the absence of active intervention limits the interpretation of this finding, which should be cautiously contextualized. RANTES levels also decreased significantly after the observation period (*p* = 0.0450). RANTES is a chemokine involved in T-cell activation and leukocyte recruitment. This reduction could indicate a modest decline in immune cell signaling, potentially related to developmental changes or non-specific effects. These results suggest a baseline trend towards decreased immune activation independent of specific dietary or probiotic interventions. Alternatively, they may reflect the natural fluctuations in immune biomarkers in children with ASD, emphasizing the importance of including a control group for comparison with active interventions. These findings highlight the need for longitudinal immune monitoring in ASD studies to distinguish treatment-specific effects from physiological variability or time-related changes.

**Figure 4:**
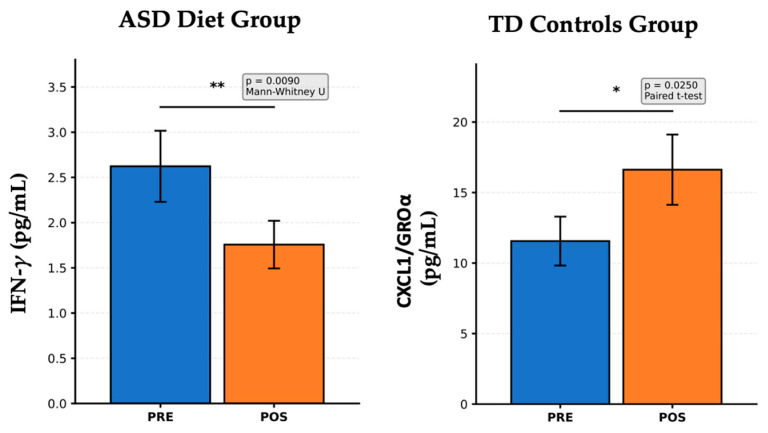
Cytokine and chemokine modulation following anti-inflammatory diet in ASD and natural variation in TD controls. Statistical markers were interpreted as * *p* < 0.05, ** *p* < 0.01.

Figure 4 illustrates cytokine and chemokine levels measured before and after intervention in two distinct groups: left panel; right panel. Children with ASD receiving an anti-inflammatory diet (left panel) show a significant reduction in IFN-γ levels after the dietary intervention (*p* = 0.0090, Mann–Whitney U test). IFN-γ is a pro-inflammatory Th1 cytokine that is implicated in chronic inflammation and immune dysregulation in ASD. This finding suggests an effective immunomodulatory response to the anti-inflammatory diet, potentially contributing to a reduction in neuroinflammatory processes, which aligns with previous reports showing that diets rich in antioxidants, omega-3 fatty acids, and fiber can reduce systemic inflammation in populations with ASD. In typically developing (TD) controls not subjected to dietary or probiotic interventions (right panel), there was a significant increase in CXCL1/GROα levels after the follow-up period (*p* = 0.0250, paired *t*-test). CXCL1/GROα is a chemokine involved in neutrophil recruitment and epithelial signaling. Since no specific intervention was applied to this group, this increase may reflect natural developmental immune variation or environmental influences unrelated to treatment. These fluctuations highlight the importance of including TD controls in longitudinal designs to distinguish between the true intervention effects and background variability. The anti-inflammatory diet in children with ASD led to a marked decrease in IFN-γ, indicating reduced Th1-mediated inflammation, which is a promising sign of dietary impact on immune function. In contrast, the increase in CXCL1/GROα in TD controls underscores the normal cytokine variability over time in typically developing children. These findings support the role of nutritional interventions in modulating inflammatory markers in ASD and reinforce the importance of control groups in interpreting changes due to treatment versus natural variation. In TD Control children with the *NeuroGutPlus* diet, no statistically significant differences were found when comparing the levels of cytokines PRE and POST. The absence of statistically significant differences in cytokine levels between the PRE and POST phases in the TD Control children receiving the *NeuroGutPlus* diet likely reflects the stability and homeostatic balance of the immune system in typically developing (TD) individuals. In healthy children without underlying neuroimmune dysregulation, baseline levels of circulating cytokines are generally within physiological ranges and tightly regulated by feedback mechanisms involving both innate and adaptive immunity.

**Figure 5:**
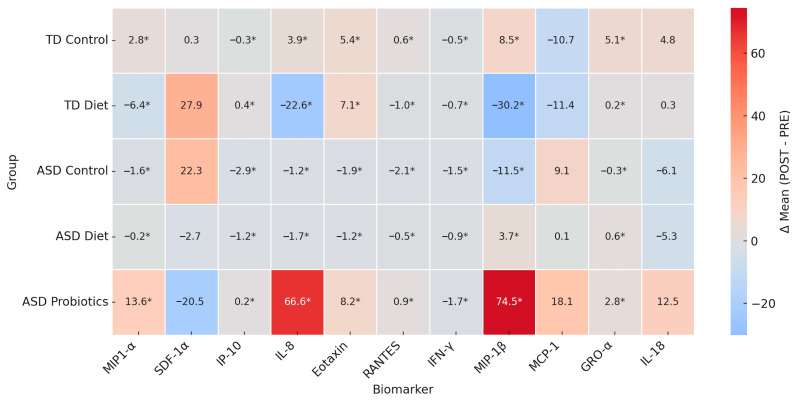
Changes in cytokine levels (Δ POST–PRE) were statistically significant (* *p* < 0.05).

To evaluate intervention effects across all groups (TD Control, TD Diet, ASD Control, ASD Diet and ASD Probiotics), a heatmap of the POST–PRE differences (Δ) were constructed from the means obtained of cytokines (MIP-1α, SDF-1α, IP-10, IL-8, Eotaxin, RANTES, IFN-γ, MIP-1β, MCP-1, GRO-α, IL-18). The asterisk (*) indicates statistical significance based on reported *p*-values (<0.05). Red represents a POST-intervention increase, and blue represents a decrease (Figure 5).

The mean difference heatmap (Δ POST–PRE) indicated that the TD Control group showed statistically significant, though modest, changes in cytokines such as MIP-1α (+2.8), IL-8 (+3.9), GRO-α (+5.1), and MIP-1β (+8.5) and a reduction in MCP-1α (−10.7) and IP-10 (−0.3). These changes could reflect baseline variability or placebo effects since there was no active intervention. In the TD Diet group, there was a significant decrease in IL-8 (−22.6) and MIP-1β (−30.2), both of which are involved in neutrophil and monocyte chemotaxis, suggesting a possible anti-inflammatory effect of the diet. In the ASD Control group, there were significant and slight decreases in several cytokines, such as IP-10, RANTES, IFN-γ, and MIP-1β, which may be related to the natural course of the disease or the placebo effect. Increased MCP-1 (+9.1) levels, a marker of monocytic activation, may indicate persistent inflammation in the absence of intervention. The diet ASD group did not show considerable increases, but there were discrete and significant decreases in SDF-1α, IL-8, Eotaxin, RANTES, IFN-γ, and MIP-1β, suggesting a general trend towards a descending immune modulation, although not as strong as in the group with probiotics. Finally, in the ASD Probiotic group, there were significantly and markedly increased levels of IL-8 (+66.6), MIP-1β (+74.5), and MCP-1 (+18.1), indicating controlled pro-inflammatory immune activation, an increase in GRO-α (+2.8) and IL-18 (+12.5), and a marked decrease in SDF-1α (−20.5). These results suggest that the probiotic intervention was able to activate specific immune signaling pathways, possibly associated with restoration of the microbiota and immune tone, rather than a simple suppression of inflammation.

Based on the magnitude and significance of these changes, candidate biomarkers for follow-up or future validation are presented in Table 3.

### 4.4. Principal Component Analysis (PCA) and Network Dynamics

Principal component analysis (PCA) was applied to PRE- and POST-intervention immune profiles to evaluate how dietary interventions (*NeuroGutPlus* anti-inflammatory diet and probiotic supplements) modify the immune profiles of children with ASD by comparing the variability and grouping of data before (PRE) and after (POST) the intervention in different study groups. Mean concentrations of 11 immunological biomarkers (cytokines and chemokines: MIP-1α, SDF-1α, IP-10, IL-8, Eotaxin, RANTES, IFN-γ, MIP-1β, MCP-1, GRO-α, IL-18) were included for the study groups TD Control (typically developing children without intervention), TD Diet (typically developing children with diet), ASD Control (children with ASD without intervention), ASD Diet (ASD children with diet), and ASD Probiotics (children with ASD with probiotics).

**Figure 6:**
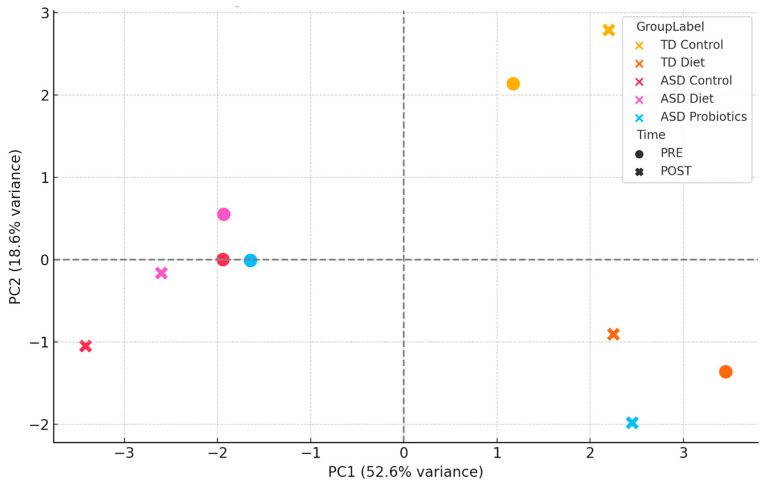
PCA clustering of immune profiles: PRE- and POST-intervention. *PC1* captures the main axis of immunological variability, separating groups with high systemic cytokine activation (e.g., ASD Probiotic POST) from those with stable or regulated immune profiles (e.g., TD Control and ASD Diet). *PC2* reflects secondary patterns, likely related to subtle differences in cytokine balance and specific immune signaling pathways. Each point represents the global immune profile of a group at a specific time (PRE or POST), with the color indicating the group and shape distinguishing the time point. The relative positions of the points illustrate immunological similarities or divergences across conditions.

During the standardization stage, variables were transformed using z-scores (mean = 0, standard deviation = 1) to correct for scale heterogeneity. To construct the PCA model, the first two main components (PC1 and PC2) were extracted, which explained most of the total variance. The first two principal components accounted for 78.6% of the total variance (PC1: 56.2%; PC2: 22.4%), justifying the use of a two-dimensional representation to capture major immunological differences between groups and intervention stages. Finally, for visual clustering, a PCA scatter plot labeled by group and temporal intervention (PRE/POST) was constructed to observe immunological dynamics (Figure 6).

These results also underscore the importance of personalized nutrition in neurodevelopmental disorders. The combined PCA of pre- and post-intervention immunological profiles showed that TD Control samples clustered tightly, indicating minimal change over time. The TD Diet and ASD Control groups showed slight changes between the PRE and POST phases. In contrast, the ASD Diet and especially the ASD Probiotic groups exhibited a notable shift between their PRE and POST states, indicating a relevant modification of the immune profile induced by the intervention. The ASD Diet group values remained close between PRE and POST, suggesting a regulatory or stabilizing effect on immune variability. This supports the hypothesis that anti-inflammatory diets can modulate the immune system without inducing overactivation while maintaining homeostasis. The ASD Probiotic POST group moved considerably away from its PRE state and from the other groups, suggesting a profound immune reorganization, likely mediated by changes in the microbiome. PCA-based clustering indicates that dietary interventions may have a stabilizing effect on immune profiles, reducing inter-individual variability. Furthermore, it shows that the interventions have differentiated effects on the immune system, where the probiotics induce greater dispersion and reorganization, while the diet seems to have a less divergent modulating effect, close to the baseline state. The inclusion of PRE and POST phases in the PCA provides a dynamic view of the immune response, which is relevant for evaluating the efficacy and safety of interventions in ASD.

## 5. Discussion

Autism spectrum disorder (ASD) is a complex neurobiological and neuropsychiatric condition that affects children’s social, communicative, and behavioral development. It is increasingly recognized as a neurodevelopmental condition with a strong immunoinflammatory component [9,30]. Chronic low-grade inflammation has been implicated in the pathophysiology of ASD, affecting behavioral, gastrointestinal, and neurocognitive domains [31]. The growing prevalence of ASD in Colombia and worldwide has led to an increase in the number of studies on its causes and management strategies. In this study, ASD was considered as an entity with an immunoinflammatory, multifactorial basis, with implications in the central nervous, gastrointestinal, and immune systems.

In this context, the application of an anti-inflammatory diet has emerged as a potentially beneficial strategy for the comprehensive care of children with ASD. Regarding interventions for children with ASD, there are several published studies whose objective is to find the best treatment for the core symptoms of the condition. In 2022, Siafis et al. published a meta-analysis of pharmacological interventions and dietary supplements for the treatment of ASD and found that aripiprazole, atomoxetine, bumetanide, and risperidone in children and adolescents with ASD, as well as fluoxetine, fluvoxamine, oxytocin, and risperidone in adults with the condition, could improve at least one domain of core symptoms. Additionally, their results show indications of improvement with carnosine, haloperidol, folic acid, guanfacine, fatty acids such as omega-3, probiotics, sulforaphane, tideglusib, and valproate, but were imprecise due to limited and statistically insignificant data [18,32].

Our findings are consistent with recent systematic reviews and meta-analyses emphasizing the therapeutic potential of microbiota-targeted interventions in ASD. Soleimanpour et al. (2024) demonstrated that probiotics exert strain-specific effects on gastrointestinal symptoms and inflammatory markers, with multi-strain formulations showing greater efficacy than single-strain products [28]. Similarly, Zeng et al. (2024) reported that probiotic supplementation alleviates gastrointestinal symptoms in children with ASD, although effects on core behavioral features remain limited [33]. These observations echo prior analyses by Mihailovich (2024), which underscore the heterogeneity of probiotic responses and advocate for personalized approaches based on immune and metabolomic profiles [34]. Beyond probiotics, Fang et al. (2025) highlighted that anti-inflammatory dietary strategies can attenuate systemic inflammation by modulating gut microbial ecology and short-chain fatty acid (SCFA) pathways [23]. Complementary evidence from Jiang and Li (2025) supports the role of early-life dietary patterns—rich in whole, fiber-dense, and minimally processed foods—in promoting microbial diversity and neurodevelopmental health [35]. However, as Agrawal et al. (2025) noted, the methodological quality of current reviews on dietary and probiotic interventions in pediatric ASD remains generally low, underscoring the need for rigorous, well-controlled trials [36]. Collectively, these converging lines of evidence reinforce the rationale for integrated dietary–probiotic strategies as feasible, non-pharmacological interventions to mitigate immune dysregulation in ASD.

Evidence indicates that diet plays a central role in regulating chronic inflammation. Nutrition alters the composition of the gut microbiota and influences brain function and neurodevelopment disorders and, in this context, the immune system plays a particularly important role in the neuroinflammation associated with ASD. One of the characteristics of children with ASD is a limited diet (recognized as one of the most effective regulators of the gut microbiota) and rejection of certain food groups, with the intake of vegetables, fruits, and proteins being lower than that of typically developing children [37]. This means that our anti-inflammatory dietary approach prioritizes foods known to improve intestinal transit—namely, those providing adequate amounts of insoluble fiber and essential micronutrients. Scientific evidence supports the anti-inflammatory properties of several vitamins and minerals, whose intake and contribution were evaluated in the present study.

Dietary intervention emphasizes whole, minimally processed foods, with increased intake of fiber, omega-3 fatty acids, and key micronutrients (e.g., zinc, magnesium, folate, and vitamins A, C, and E), all of which have known anti-inflammatory or immunoregulatory roles. A diet low in gluten and FODMAPs further aligns with clinical insights into dietary contributors to intestinal permeability and inflammation in ASD [38,39]. Therefore, our *NeuroGutPlus* anti-inflammatory diet emerges as a promising approach to addressing metabolic issues and meeting the nutritional needs of children, while reducing symptoms and comorbidities associated with autism. Thus, adequate supplementation may play an important role in ASD treatment. The *NeuroGutPlus* diet was designed based on recommendations from RIEN and TCAC to ensure a balanced intervention. Improvements in BMI, body composition, and gastrointestinal symptoms have been reported, indicating the efficacy of personalized nutritional strategies.

Our goal was to reduce systemic inflammation, balance the microbiota, and decrease digestive symptoms, which could mitigate oxidative stress and intestinal permeability associated with ASD. There is evidence of a high prevalence of gastrointestinal problems, as established by the Rome IV criteria, in children with autism. A possible connection between gastrointestinal function and the expression of autistic symptoms is a need for research to propose possible strategies to improve this condition. Evidence also supports a two-way connection between the central and gastrointestinal systems. Alterations in gastrointestinal function could influence the expression of autistic symptoms and vice versa. In this context, the implementation of an anti-inflammatory diet is presented as a well-founded strategy to address both the challenges associated with autism and secondary gastrointestinal problems [40].

In recent years, increasing attention has been paid to the relationship between diet and gastrointestinal dysfunction in children with autism, since gastrointestinal problems are common in this population and may be linked to dietary factors. The need for an objective approach that allows for correct medical and nutritional management allows the development of parameters based on scientific evidence. In the case of functional gastrointestinal disorders, the Rome IV criteria should be used in the international pediatric guidelines. Analysis of the determinants of gastrointestinal disorders helps clarify the role of diet as a trigger or enhancer of symptoms [41]. The integration of the Rome IV criteria for gastrointestinal symptom evaluation is a strength of this study, as it ensures consistency with international standards and enhances clinical interpretability. Our results reaffirm the high prevalence of functional gastrointestinal disorders in ASD and their likely association with microbiota dysbiosis and dietary patterns [42]. In this context, our anti-inflammatory diet represents a feasible and evidence-based approach to reduce intestinal inflammation, modulate immune activity, and potentially improve the core and associated ASD symptoms.

Improvements in anthropometric outcomes and gastrointestinal symptoms support previous studies highlighting the benefits of tailored diets on both gut function and behavior in ASD. The observed effects may be partly explained by gut–brain axis modulation, wherein the gut microbiota communicates with the central nervous system via immune, endocrine, and neural pathways. This bidirectional crosstalk has become a central focus of ASD research, particularly in dietary and microbiota-based interventions [43,44].

Recent studies support the use of multi-strain probiotics in pediatric populations with ASD and gastrointestinal disturbances, showing benefits in behavioral regulation (reduced anxiety and improved focus), improvement of persistent gastrointestinal symptoms, decrease in systemic inflammatory markers, and synergistic potential when combined with anti-inflammatory or fiber-rich diets [28,45]. With the administration of probiotic supplementation in this experimental group, we sought to enhance immune and intestinal modulation without altering the child’s usual base diet, facilitating comparison with other nutritionally intervened groups (GE1 and GE2). This strategy allowed for the analysis of the specific effects of probiotics on immunological variables.

Both the anti-inflammatory diet and probiotics showed favorable immunomodulatory effects in children with ASD, as evidenced by the decrease in IFN-γ, a key cytokine associated with neuroinflammatory processes. The probiotic group also showed an increase in IL-8 and MIP-1β, suggesting a possible beneficial activation of the innate immune system in the context of microbial and metabolic reprogramming. The ASD Control group also showed decreased levels of pro-inflammatory cytokines. This could be attributed to natural biological variations, maturation of the immune system, or uncontrolled effects (e.g., general diet and family environment), highlighting the importance of using controlled designs and close monitoring. TD controls presented variations in chemokines, supporting the hypothesis that there are immunological fluctuations in children without ASD, which should be considered when interpreting the effects of interventions. Taken together, our results suggest that nutritional interventions (probiotics and anti-inflammatory diets) can favorably modulate the immune system in children with ASD, with potential indirect clinical benefits [46,47]. These findings justify the expansion of future studies to include larger sample sizes, parallel clinical measures, and longitudinal follow-up. Although both interventions showed beneficial effects, the broader and more consistent immunological shifts observed with *NeuroGutPlus* suggest that diet may have a more systemic effect than probiotics alone. Nonetheless, combining both strategies could offer synergistic benefits, as supported by emerging clinical trials that explore multimodal interventions. Our findings reinforce the role of immune dysregulation in ASD and support the hypothesis that dietary modulation exerts systemic immunological effects. While probiotics offer some benefits, *NeuroGutPlus* demonstrated a broader and more potent impact on the cytokine and chemokine milieu. The observed decline in pro-inflammatory markers is particularly significant given the association of chronic low-grade inflammation with ASD symptomatology, potentially ameliorating neuroinflammation.

Our results reinforce this perspective, demonstrating that both an anti-inflammatory diet (*NeuroGutPlus*) and multi-strain probiotics exert favorable immunomodulatory effects in children with ASD, notably by modulating circulating cytokines and chemokines. Scientific evidence suggests that chronic inflammation may play a significant role in autism symptomatology, including behavior, communication, and social skills.

The results of this study suggest that an anti-inflammatory diet should be integrated as a public health strategy for the ASD population, tailored to local contexts and population needs and a differential approach. Immunological and nutritional tests should be included within the standard clinical follow-up as well as training in precision medicine for professionals. Finally, we consider it important to evaluate the economic and social impact of interventions in the ASD population.

In conclusion, our results show that both the *NeuroGutPlus* anti-inflammatory diet and probiotic interventions attenuate the Th1-type immune response, which is frequently dysregulated in patients with ASD. The probiotic intervention shows a more heterogeneous profile, with a selective increase in certain chemokines (such as MIP-1β) possibly associated with the reconfiguration of the gut microbiota, while the anti-inflammatory diet, although more stable in terms of chemokines, has more consistent effects on reducing key inflammatory mediators, particularly those linked to the chronic activation of the immune system. Both interventions showed specific immunomodulatory effects, with the diet being more uniform in its anti-inflammatory effect, while probiotics generated a more variable but equally promising response. These results support the use of nutritional and microbial strategies as viable non-pharmacological interventions in the clinical treatment of ASD.

This study had several limitations. The relatively small sample size limited both the statistical power and the generalizability of the findings. No formal power calculation was conducted because the primary objective was hypothesis generation and mechanistic insight rather than confirmatory analysis. Future investigations should employ adequately powered, multi-center designs integrating clinical and behavioral endpoints to validate and extend these findings.

Another limitation of this study is the absence of behavioral outcome integration. Although clinical follow-up visits included observational assessments, variability in therapeutic regimens and lack of standardized baseline instruments precluded meaningful correlation with immunological markers. This approach was intentional to preserve internal validity and focus on mechanistic immune insights, a critical gap in ASD intervention research. Future studies should incorporate validated behavioral and neuropsychological assessments alongside immune biomarkers to elucidate clinically significant relationships between systemic inflammation and behavioral phenotypes.

From a public health perspective, our findings support the inclusion of nutritional and immunological assessments in the routine management of ASD, particularly in resource-constrained settings, where pharmacological treatments may be limited. A culturally and regionally tailored dietary intervention, such as *NeuroGutPlus*, could serve as a cost-effective, non-pharmacological strategy to reduce the inflammatory burden and improve the quality of life in children with ASD.

## 6. Conclusions

This study provides evidence that a structured anti-inflammatory diet such as *NeuroGutPlus* exerts a more comprehensive immunomodulatory effect than probiotics alone in children with ASD. Targeting systemic inflammation through dietary strategies may represent a viable adjunctive approach for managing immune-related dysfunction in ASD.

## Figures and Tables

**Table 1:** Intragroup comparison of baseline levels of immune biomarkers in children with and without ASD before nutritional interventions.

*Biomarkers*	*Test Applied*	*Mean* *TD Control Group*	*Mean TD DIET Group*	*Mean ASD Control Group*	*Mean ASD Diet Group*	*Mean ASD Probiotics Group*	*p-Value*
MIP-1α	Kruskal–Wallis	10.080	16.832	5.090	4.569	3.707	0.401
SDF-1α	Kruskal–Wallis	946.281	869.595	802.231	865.991	863.594	0.103
IP-10	ANOVA	10.582	15.114	8.735	8.151	8.148	0.222
IL-8	Kruskal–Wallis	10.706	33.512	3.244	6.347	2.934	0.798
Eotaxin	Kruskal–Wallis	24.292	36.321	24.400	22.750	28.053	0.161
RANTES	Kruskal–Wallis	12.456	14.039	9.985	9.660	10.470	0.145
IFN-γ	Kruskal–Wallis	3.583	3.459	2.952	2.623	3.324	0.138
MIP-1β	Kruskal–Wallis	154.583	173.825	117.553	121.844	125.071	0.160
MCP-1	ANOVA	50.806	91.573	39.022	36.012	44.843	0.004
GRO-α	Kruskal–Wallis	11.559	8.030	8.040	6.261	9.150	0.364
IL-18	Kruskal–Wallis	23.720	19.613	21.067	23.305	10.084	0.183

**Table 2:** Intragroup comparison of immune biomarker levels in children with and without ASD after nutritional interventions.

*Biomarkers*	*Test Applied*	*Mean* *TD Control Group*	*Mean TD DIET Group*	*Mean ASD Control Group*	*Mean ASD Diet Group*	*Mean ASD Probiotics Group*	*p-Value*
MIP-1α	Kruskal–Wallis	12.894	10.419	3.529	4.367	17.309	0.003
SDF-1α	ANOVA	946.593	897.535	824.535	863.296	843.127	0.504
IP-10	Kruskal–Wallis	10.318	15.542	5.825	6.929	8.361	0.007
IL-8	Kruskal–Wallis	14.585	10.874	2.057	4.637	69.528	0.00003
Eotaxin	Kruskal–Wallis	29.742	43.466	22.457	21.594	36.272	0.010
RANTES	ANOVA	13.017	13.038	7.872	9.173	11.401	0.006
IFN-γ	Kruskal–Wallis	3.068	2.736	1.496	1.756	1.605	0.03
MIP-1β	Kruskal–Wallis	163.120	143.668	106.039	125.589	199.603	0.001
MCP-1	Kruskal–Wallis	40.101	80.125	48.100	36.085	62.974	0.06
GRO-α	Kruskal–Wallis	16.622	8.193	7.788	6.839	11.925	0.017
IL-18	Kruskal–Wallis	28.472	19.920	14.997	17.958	22.541	0.344

**Table 3:** Cytokines that were significantly modulated by dietary and probiotic interventions in ASD and TD groups.

Cytokine	Relevance	Group(s)	Exchange Rate
**MIP-1** **β**	Involved in inflammation and chemotaxis	ASD Probiotics (+74.5), TD Diet (−30.2)	↑/↓
**IL-8**	Neutrophilia, intestinal inflammation	ASD Probiotics (+66.6), TD Diet (−22.6)	↑/↓
**MCP-1**	Monocytic activation	ASD Probiotics (+18.1), ASD Control (+9.1)	↑
**SDF-1α**	Immune homeostasis, cell migration	ASD Probiotics (−20.5), ASD Diet (−2.7)	↓

## Data Availability

The raw data are available upon request from the corresponding author.
